# Relationships between depression, anxiety, type D personality, and worry and rumination in patients with coronary heart disease

**DOI:** 10.3389/fpsyg.2022.929410

**Published:** 2022-09-14

**Authors:** Kristoffer Tunheim, Toril Dammen, Silje Baardstu, Torbjørn Moum, John Munkhaugen, Costas Papageorgiou

**Affiliations:** ^1^The Medical Faculty, Institute of Clinical Medicine, University of Oslo, Oslo, Norway; ^2^Department of Medicine, Drammen Hospital, Vestre Viken Trust, Drammen, Norway; ^3^Division of Mental Health and Addiction, Department for Research and Innovation, Oslo University Hospital, Oslo, Norway; ^4^Department of Child Health and Development, Norwegian Institute of Public Health, Oslo, Norway; ^5^Department of Behavioral Medicine, The Medical Faculty, University of Oslo, Oslo, Norway; ^6^Department of Psychology, University of Oslo, Oslo, Norway; ^7^Priory Hospital Altrincham, Cheshire, United Kingdom

**Keywords:** anxiety, depression, type D personality, worry, rumination, metacognition, metacognitive therapy, coronary heart disease

## Abstract

Psychological distress, including depression and anxiety, and Type-D personality are prevalent in patients with coronary heart disease (CHD) and associated with poor cardiovascular outcomes. Worry and rumination may be among the core features responsible for driving psychological distress in these patients. However, the nature of associations between these constructs remains to be delineated, yet they may have implications for the assessment and treatment of CHD patients. This study aimed to (1) explore the factorial structure and potential overlap between measures of depression, anxiety and the Type-D personality factors known as negative affectivity and social inhibition, and (2) examine how these constructs relate to worry and rumination in a sample of 1,042 CHD outpatients who participated in the in the cross-sectional NORwegian CORonary Prevention study. We conducted confirmatory factor analyses (*n* = 1,042) and regression analyses (*n* = 904) within a structural equation modeling framework. Results showed all constructs to have acceptable factor structure and indicated an overlap between the constructs of depression and negative affectivity. Worry was most strongly associated with anxiety, whereas rumination was most strongly associated with depression and negative affectivity. The results suggest conceptual similarities across the measures of depression and negative affectivity. They further suggest that intervention efforts could benefit from targeting worry and/or rumination in the treatment of CHD outpatients presenting with symptoms of psychological distress.

## Introduction

Coronary Heart Disease (CHD) is the leading cause of death worldwide (Finegold et al., 2013). Type D personality (TDP) and depression have been recognized as potential risk factors for the development of cardiac disease as well as for poor cardiovascular prognosis in CHD patients (Kupper and Denollet, 2018). Anxiety is also associated with adverse outcomes in this population (Roest et al., 2010; Tully et al., 2014). However, despite the high prevalence (30–40%) of clinically significant symptoms of depression and anxiety in CHD patients (Reid et al., 2013), the effectiveness of psychological treatment of these symptoms in CHD patients is generally poor, with extant research reporting small effect sizes of treatment on symptoms and no effect on cardiac prognosis (Richards et al., 2017). Therefore, with the purpose of developing more effective psychological treatment methods for this specific patient group, there is a definite need to gain more knowledge about the interrelationship between factors of psychological distress as well as identifying the key factors to be targeted in the treatment of such distress. Worry and rumination may represent two such key factors as they are important components in a more recent theoretically and empirically grounded model for the effective treatment of depression and anxiety (i.e., the metacognitive model; Wells, 2009).

The most recent European clinical practice guidelines for cardiovascular disease (CVD) prevention recommend screening for depression, anxiety and TDP in patients with CHD as these factors are associated with unhealthy lifestyle and poor adherence to treatment and participation in cardiac rehabilitation programs (Visseren et al., 2021). TDP is conceptualized as the combination of high levels of negative affectivity (NA)—the tendency to experience negative emotions across time and situations, including feelings of dysphoria, worry, and tension—and high levels of social inhibition (SI)—the tendency to inhibit self-expression in social interactions and to avoid negative reactions from others (Denollet, 2005). More recently, however, the prognostic role of TDP has been disputed, partly because it has been argued that it overlaps with the construct of depression (Ossola et al., 2015). Furthermore, studies on the prognostic significance of TDP as an independent risk factor for poor cardiovascular prognosis when controlling for depression (Doyle et al., 2011; Starrenburg et al., 2013) and anxiety have reported inconsistent results (Smaardijk et al., 2020). Thus, it has been suggested that the effect of TDP on cardiovascular outcomes may be due to its similarities with depression, which in turn, has led many to question the independent role of TDP for cardiovascular prognosis in CHD patients (Conden et al., 2017).

Whilst some have argued that TDP and depression may be similar or overlapping constructs (Lespérance and Frasure-Smith, 1996), others have suggested that TDP refers to a more covert form of distress that is distinct from depression (Denollet and Pedersen, 2008). The former suggestion is based on the observation of high prevalence of previous and current significant depression symptoms or depressive disorders among patients with TDP (Bergvik et al., 2010; Christodoulou et al., 2013; Starrenburg et al., 2013; Conden et al., 2014). Moderate to strong correlations between the negative affectivity factor of TDP and anxiety have also been found (Kudielka et al., 2004; Pelle et al., 2009; Bergvik et al., 2010; Svansdottir et al., 2012). Together, these findings indicate that there might be conceptual similarities between the constructs of depression, anxiety and negative affectivity that could be accounted for by a common underlying denominator. As pointed out by Suls (2018), who reviewed studies assessing the distinctiveness and overlap of depression, anxiety, anger, and negative affectivity, these constructs appear to exhibit both construct and measurement overlap. However, as other studies have not found such associations (Denollet et al., 2009), this issue is still controversial and needs to be empirically explored. Such knowledge is important as it may have implications for the development of new psychological treatment methods aimed at CHD patients with psychological distress and/or TDP. In summary, the current literature suggests that Type D personality, depression, and anxiety may play an important role in risk and prognosis of CHD. However, there have been inconsistent data on the overlap and distinctiveness between the measures aimed at assessing these constructs. In addition, the processes of worry and rumination are thought to play an important role in the development and maintenance of depression and anxiety although their relationships with anxiety, depression, and Type D personality in CHD remain largely unexplored.

One way of elucidating the overlap and distinctiveness of depression, anxiety and TDP is to apply factor analyses. To date, four previous studies have explored such potential overlap (i.e., shared variance) and similarities between depression, anxiety and TDP using the Hospital Anxiety and Depression Scale (HADS; Zigmond and Snaith, 1983) and the Type D Scale (DS14; Denollet, 2005) by applying different factor analytic approaches. Pelle et al. (2009), Kudielka et al. (2004), and Svansdottir et al. (2012) applied exploratory factor analysis and found little or no overlap between the negative affectivity and social inhibition scales of DS14 and the depression and anxiety scales of HADS. Ossola et al. (2015), however, combined exploratory factor analysis with a partial confirmatory factor analysis (pCFA) and found substantial overlap between depression and the negative affectivity scale of TDP. Interestingly, all these studies found little or no shared variance between the TDP scales negative affectivity and social inhibition, which suggests that these two scales may represent distinct and independent constructs. To our knowledge, only two studies have explored similarities and differences between depression, anxiety, negative affectivity, and social inhibition using CHD samples with acute coronary syndrome (ACS) (Pelle et al., 2009; Ossola et al., 2015), or without depression (Ossola et al., 2015). Thus, studies assessing such hypotheses using samples of outpatients with chronic CHD including those with depression are lacking. We applied the HADS for comparative purposes. Furthermore, HADS is commonly used to identify depression in CHD patients (Thombs et al., 2016).

There is clearly a need for effective treatment of psychological distress, such as depression and anxiety, in CHD patients (Richards et al., 2017). Metacognitive therapy (MCT) is a transdiagnostic psychological treatment, which is grounded in the metacognitive model for the understanding of the development and maintenance of psychological distress and disorders (Wells, 2009). A key goal of MCT is to target two core features that may be responsible for driving psychological distress, i.e., worry and rumination. Worry is defined as a chain of negative thoughts that are predominantly verbal in content and aimed at problem-solving (Borkovec et al., 1983). Rumination involves “repetitively focusing on the fact that one is depressed; on one’s symptoms of depression; and on the causes, meaning, and consequences of depressive symptoms” (Nolen-Hoeksema, 1991, p. 569). Furthermore, the tripartite model of anxiety and depression posits that anxiety and depression often occur together due to shared genetic factors, as well as a common distress factor (Clark and Watson, 1991).

Despite the potential benefit of targeting worry and rumination in the treatment of psychological distress, it remains to be explored how these two constructs relate to anxiety, depression, and TDP in CHD patients. Such knowledge may have implications both for the development of screening tools for assessing a psychological distress in patients with CHD as well as for developing and providing more effective treatment of such distress for these patients.

The first aim of the present study was to explore the factor structure and potential similarities and differences between the constructs of depression, anxiety, negative affectivity, and social inhibition using confirmatory factor analyses. As studies empirically testing the interrelationship between these factors in chronic CHD patients are lacking, we adopted an exploratory approach in generating the hypotheses based on a combination of *a priori* theory and the small body of existing empirical evidence in other samples. First, based on previous findings (Suls, 2018), we wanted to explore the degree of overlap between the anxiety and depression scales of the HADS instrument and the negative affectivity scale of the DS14 instrument. Based on the findings of Ossola et al. (2015), we also aimed to explore the degree of overlap between the depression scale of the HADS and the negative affectivity scale of the DS14. Furthermore, based on a study indicating strong correlations between negative affectivity and anxiety (Bergvik et al., 2010), we wanted to explore potential overlap between the anxiety scale of the HADS and the negative affectivity scale of the DS14.

The second aim of the study was to examine how worry and rumination would be associated with depression, anxiety and the TDP factors among CHD patients. Given the lack of empirical evidence in the literature concerning the nature of such associations, we also adopted an exploratory approach in generating our hypotheses. In this respect, we were only able to assume that the factors of worry and rumination would be significantly and positively associated with all scales of the HADS and the DS14 instruments. If so, there might be significant implications for the future treatment of symptoms of depression, anxiety and/or personality dimensions in these patients.

In summary, the current study aimed to: (1) explore the factor structure and the potential similarities and overlap between measures typically used to assess psychological distress and personality in CHD patients, including the HADS and the DS14 and (2) examine how these constructs are associated with worry and rumination.

## Materials and methods

### Design and population

**FIGURE 1:**
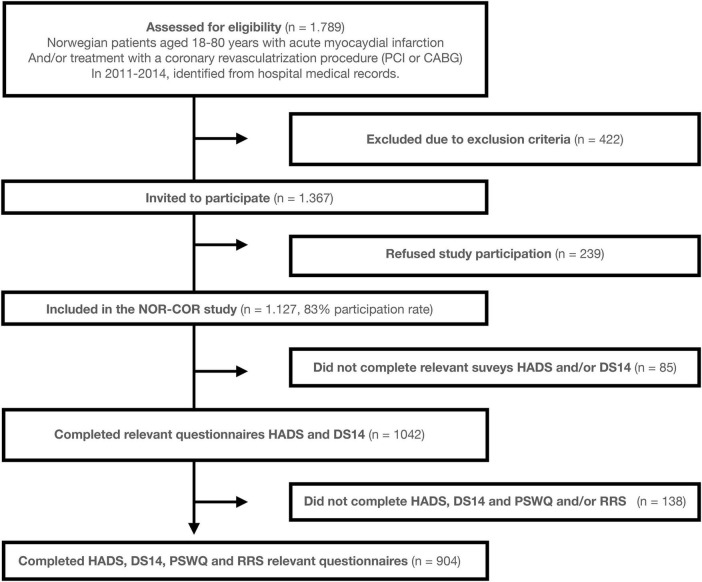
Study flow chart. The NOR-COR Study, The Norwegian Coronary Prevention Study; HADS, Hospital Anxiety and Depression Scale; DS14, The Type D Scale; PSWQ, Penn State Worry Questionnaire; RRS, Ruminative Response Scale.

A cross-sectional study (Munkhaugen et al., 2016) conducted in 2014–2015 included 1,127 patients with a CHD event 2–36 (median 16) months earlier (i.e., between 2011 and 2014). The following inclusion criteria were applied: aged 18–80 years with a first or recurrent CHD event, which was defined as acute type 1 myocardial infarction and/or a revascularization procedure (coronary artery bypass grafting or percutaneous coronary intervention). The exclusion criteria were: not being able to understand the Norwegian language, cognitive impairment including living in nursing homes, psychosis, drug abuse, short life expectancy due to terminal heart (NYHA class 4), lung disease, liver disease, kidney disease (stage 5), or malignant disease. Eighty-five participants that did not complete either HADS or DS14 were excluded from the current study. The study flow chart is illustrated in Figure 1.

Participants were recruited from Drammen and Vestfold hospitals, which in total have a catchment area of 7.4% of the Norwegian population (380,000 inhabitants). This sample represents a blend of urban and rural districts and is representative of education, economy, age distribution, morbidity, and mortality levels in the Norwegian population (Munkhaugen et al., 2016).

The study was approved by the Regional Committee for Medical and Health Research Ethics in the South-East Region of Norway (REC South-East) (2013/1885). All patients signed a written informed consent in advance of participation.

### Measures

Information about age, gender, CHD index diagnosis, previous CHD events, time since the index event, somatic comorbidity assessed by the Charlson comorbidity score (Charlson et al., 1987) at the time of the index CHD event was collected from hospital records. Information about body weight, height, blood pressure and level of low-density lipoprotein cholesterol and c-reactive protein was collected from a clinical examination and blood samples at study inclusion in 2014-15.

A comprehensive self-report questionnaire was filled out by the CHD outpatients in this study. Symptoms of depression and anxiety were assessed using the HADS (Zigmond and Snaith, 1983). The HADS consists of 14 items, each scored on a five-point Likert scale (0–3) with higher scores indicating more severe symptoms. The HADS generates two seven-item scales, a depression scale (HADS-D) and an anxiety scale (HADS-A). The HADS has demonstrated good psychometric properties in several studies, including in CHD patients (Haddad et al., 2013). The Norwegian version of the HADS has shown good internal consistency and acceptable validity across studies (Leiknes et al., 2016). Examples of items measuring HADS-D are “I feel as if I am slowed down,” and HADS-A “I get sudden feelings of panic.” The 4-week test-retest reliabilities in the cross-sectional study were 0.92 for HADS-A and 0.94 for HADS-D (Peersen et al., 2017). In the present study, the Cronbach’s alphas were 0.84 for HADS-A and 0.76 for HADS-D.

Type-D personality (Denollet, 2005) was assessed using the DS14, which is a self-report scale consisting of two scales measuring negative affectivity and social inhibition, respectively. Each scale consists of seven questions rated on a five-point Likert scale (0–4). A person is defined as having TDP if he or she has a score =10 on both the negative affectivity and social inhibition scales (Denollet, 2005). The Norwegian version of DS14 has been validated in Norwegian cardiac patients and found to have good psychometric properties (Bergvik et al., 2010). Examples of items measuring negative affectivity are “I often feel unhappy” and for social inhibition “I am a closed kind of person.” The 4-week test-retest reliabilities were 0.91 for negative affectivity and 0.90 for social inhibition (Peersen et al., 2017). In the present study, Crohnbach’s alphas were 0.87 for negative affectivity and 0.86 for social inhibition.

Worry was measured using the Penn State Worry Questionnaire (PSWQ) (Meyer et al., 1990). This scale consists of 16 items rated on a five-point Likert scale (1–5), sum scores range from 16 to 80 where higher scores indicate a greater predisposition to worry. Examples of items of the PSWQ include “I worry all the time.” and “My worries overwhelm me.” The PSWQ has good psychometric properties (Startup and Erickson, 2006), and the 4-week test-retest reliability and the Cronbach’s alpha of the PSWQ was 0.91 (Peersen et al., 2017).

Rumination was assessed using the Ruminative Response Scale (RRS; Treynor et al., 2003). The RRS is a 22-item questionnaire that is rated on a four-point Likert scale (1–4). The total scores range from 22 to 88 with higher scores indicating higher levels of rumination. Examples of items of the RRS include “think about how sad I feel” and “think about how hard it is to concentrate.” The test-retest reliability of the RRS was 0.88 and the Cronbach’s alpha was 0.96 (Peersen et al., 2017).

### Statistical analyses

Statistical analyses were performed within a structural equation modeling framework, using Mplus version 8.5 (Muthén and Muthén, 2017), and carried out in several steps. First, measurement models were estimated through the means of CFA by constructing latent factors for each of the scales in the study. Due to the large number of total items, as well as for of some of the individual scales (PSWQ and RRS), we applied parceling (i.e., reducing the number of indicators into parcels) as this approach is considered to provide a superior test of structural model parameters because the constructs are defined more precisely (Little et al., 2013). Parceling was conducted by constructing three parcels for each of the factors of the depression, anxiety, negative affectivity, and social inhibition scales based on their respective items, with two to three items in each parcel, and by constructing four parcels for the worry and rumination measures based on their respective items, with four to six items in each parcel. For all constructs, items were randomly assigned and evenly distributed to their respected parcels following Little et al. (2013) and mean-composite scores of the parcels were calculated thereafter. The parcels were then used as indicators of their respective latent factor. To ensure that those with missing data were excluded from the analysis, non-responders were defined both as those who did not complete or did not respond to all items on each questionnaire and they were excluded from the analysis prior to the item parceling procedure.

**FIGURE 2:**
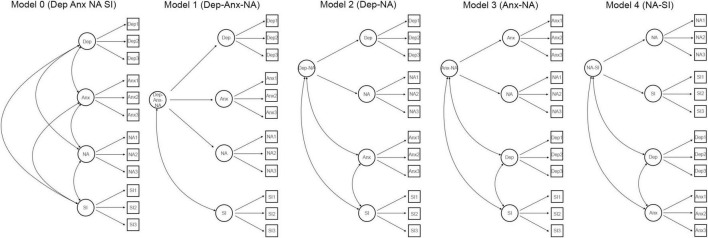
Confirmatory factor analysis models of the relationship between depression, anxiety, negative affectivity, and social inhibition. Circles represent latent variables and squares represent observed variables. Anx, Anxiety; Dep, Depression; NA, Negative Affectivity; SI, Social Inhibition.

To examine the possibility of overlap between the scales, five different models were estimated. We first estimated a baseline model (Model 0) where the latent factors of depression, anxiety, negative affectivity, and social inhibition were specified simultaneously as distinct constructs based on their respective item parcels. Based on previous theory and factor analytic studies on HADS and DS14, we then estimated four alternative models, each specified with a higher-order factor through the means of second-order CFA that was hypothesized to account for overlap between (a) the depression, anxiety and the negative affectivity latent factors (Model 1: Dep-Anx-NA); (b) the depression and negative affectivity latent factors (Model 2: Dep-NA); (c) the anxiety and the negative affectivity latent factors (Model 3: Anx-NA); and finally (d) the negative affectivity and social inhibition latent factors (Model 5: NA-SI). An illustration of the models is shown in Figure 2. Second-order CFA models are considered appropriate when the lower-order factors are highly correlated with each other and in cases where a higher-order factor is hypothesized to account for the relationship among the lower-order factors (Chen et al., 2006), as is the case in the current study. Given that these models are considered non-nested, model selection was based on the principle of parsimony and through the means of several model fit approaches, including the Akaike (AIC) and Bayesian (BIC) fit indices (i.e., smaller values indicate better model fit) (Aikake, 1973; Schwarz, 1978), as well as the standard fit indices Confirmatory Fit Indices (CFI), Tucker-Lewis Index (TLI), Root Mean Square Error of Approximation (RMSEA) and Standardized Root Mean Squared Residual (SRMR). Suggested cut-off values (Hu and Bentler, 1999) for these standard indices are SRMR close to 0.08 or below, RMSEA values close to 0.06 and below, and CFI and TLI values close to or above 0.95 are considered to indicate good fit. Finally, regression path analyses were used to explore the association between the HADS and the DS14 latent factors (i.e., anxiety, depression, negative affectivity, and social inhibition) with the worry and rumination latent factors. The Satorra-Bentler (MLM) Least Squares-estimator was used to address the non-normal nature of the data (Satorra and Bentler, 1994).

## Results

A total of 1,042 patients completed both HADS and DS14, and among these, 138 (13.2%) patients had missing data on PSWQ or RRS. Demographic and clinical data are described in Table 1. No significant differences were found for any of the clinical or psychological characteristics between the 1,127 patients included and the 1,042 responders of HADS and DS14 and the 904 responders of HADS, DS14, PSWQ, and RRS (Supplementary Table 1). Pearson’s correlations between mean-composite scores of the scales are shown in Table 2. All scales correlated significantly; negative affectivity correlated moderately/strongly with all other scales whereas social inhibition correlated weakly to moderately with all other scales. The measurement models for each of the scales showed acceptable to good fit (Supplementary Table 2: Model fit).

**TABLE 1:** Clinical characteristics of the patients.

Age, mean (SD)	61.5 (9.6)
Female gender,% (n)	20.0 (217)
Low education (<12 years),% (n)	85.7 (725)
Time since the cardiac event (months), mean (SD)	17.2 (10.5)
Acute myocaydial infarction,% (n)	79.1 (824)
Stable or unstable angina,% (n)	20.9 (218)
Previously one or more coronary event prior to the index event,% (n)	24.1 (251)
Participation in cardiac rehabilitation,% (n)	51.1 (532)
HADS-A, mean (SD)	4.7 (3.7)
HADS-D, mean (SD)	3.8 (3.2)
HADS-T, mean (SD)	8.5 (6.2)
DS14-NA, mean (SD)	7.0 (5.9)
DS14-SI, mean (SD)	7.5 (5.6)
DS14-T, mean (SD)	14.5 (10.0)
Type D personality, % (n)	18.1 (189)

HADS-T, Hospital Anxiety and Depression Scale total mean score; HADS-A, Hospital Anxiety and Depression Scale anxiety subscale mean score; HADS-D, Hospital Anxiety and Depression Scale depression subscale mean score; DS14-T, Type D Scale total mean score; DS14-SI, Type D Scale social inhibition subscale mean score; DS14-NA, Type D Scale negative affectivity subscale mean score.

**TABLE 2:** Correlations between anxiety, depression, negative affectivity, social inhibition, worry and rumination (*n* = 904).

	HADS-A	HADS- D	DS14 NA	DS14 SI	PSWQ	RRS
HADS-A		0.63*	0.73*	0.40*	0.74*	0.68*
HADS-D	0.63*		0.63*	0.49*	0.55*	0.65*
DS14 NA	0.73*	0.63*		0.53*	0.71*	0.69*
DS14 SI	0.40*	0.49*	0.54*		0.38*	0.39*
PSWQ	0.74*	0.55*	0.71*	0.38*		0.59*
RRS	0.68*	0.65*	0.69*	0.39*	0.59*	

*p < 0.001. HADS-A, Hospital Anxiety and Depression Scale anxiety subscale; HADS-D, Hospital Anxiety and Depression Scale depression subscale; DS14 NA, Type D Scale negative affectivity subscale; DS SI, Type D Scale, social inhibition subscale; PSWQ, Penn State worry questionnaire; RRS, Ruminative Response Scale.

### Exploring overlap between scales

The five *a priori* models hypothesized (Figure 2) concerning possible overlap (i.e., common variance) between the scales of the HADS and DS14 measures, including the baseline model (Model 0) and the four alternative models (Models 1–4). The results in Table 3 show that all models had an acceptable fit. The model of the depression and negative affectivity factors (Model 2: Dep-NA) showed the overall most favorable fit to the data, closely followed by the baseline model.

**TABLE 3:** Fit indices for different models in the confirmatory factor analyses and results of comparison between the baseline model and each second order model.

	χ ^2^	*df*	SRMR	RMSEA	CFI	TLI	AIC	BIC	Sample adjusted BIC	Δχ ^2^	Δ *d*	*p*
**Baseline model:**												
Model 0 Dep, Anx, NA and SI	234.4	48	0.031	0.061 [0.053–0.069]	0.97	0.96	28467.8	28675.7	28542.3			
**2nd order models**												
Model 1 Dep-Anx-NA	293.2	50	0.041	0.068 [0.061–0.076]	0.96	0.95	28534.4	28732.3	28605.3	58.8	2	< 0.05
Model 2 Dep-NA	234.6	49	0.031	0.06 [0.053–0.068]	0.97	0.96	28466.6	28669.5	28539.3	0.2	1	> 0.05
Model 3 Anx-NA	288.7	49	0.039	0.069 [0.061–0.076]	0.96	0.95	28531.5	28734.4	28604.2	54.3	1	< 0.05
Model 4 NA-SI	264.5	49	0.040	0.065 [0.057–0.073]	0.97	0.95	28500.6	280703.5	28573.3	30.1	1	< 0.05

Dep, Depression; Anx, Anxiety; NA, Negative Affectivity; SI, Social Inhibition; χ^2^, Chi-square test; df, Degrees of freedom; SRMR, Standardized Root Mean Squared Residual; RMSEA, Root Mean Square Error of Approximation; CFI, Comparative Fit Index; TLI, Tucker-Lewis Index; AIC, Akaike Information Criteria; BIC, Bayesian Information Criteria; Δχ^2^, Difference between chi-square-test from baseline model; Δd, Difference in degrees of freedom from baseline model; *p*, *p*-value.

### Associations of depression, anxiety, negative affectivity and social inhibition with worry and rumination

To explore the associations between the constructs of psychological distress and personality and the worry and rumination measures, two path regression models were generated based on the findings from the previous step. In the first model, the latent factors worry and rumination were specified as predictors of the latent factor capturing overlap between depression and negative affectivity (Dep-NA). In the second model, the latent factors worry and rumination were specified as predictors of all of the latent factors of the baseline model (CFA Model 0), that is, all the constructs of psychological distress (anxiety and depression) and personality (negative affectivity and social inhibition).

**FIGURE 3:**
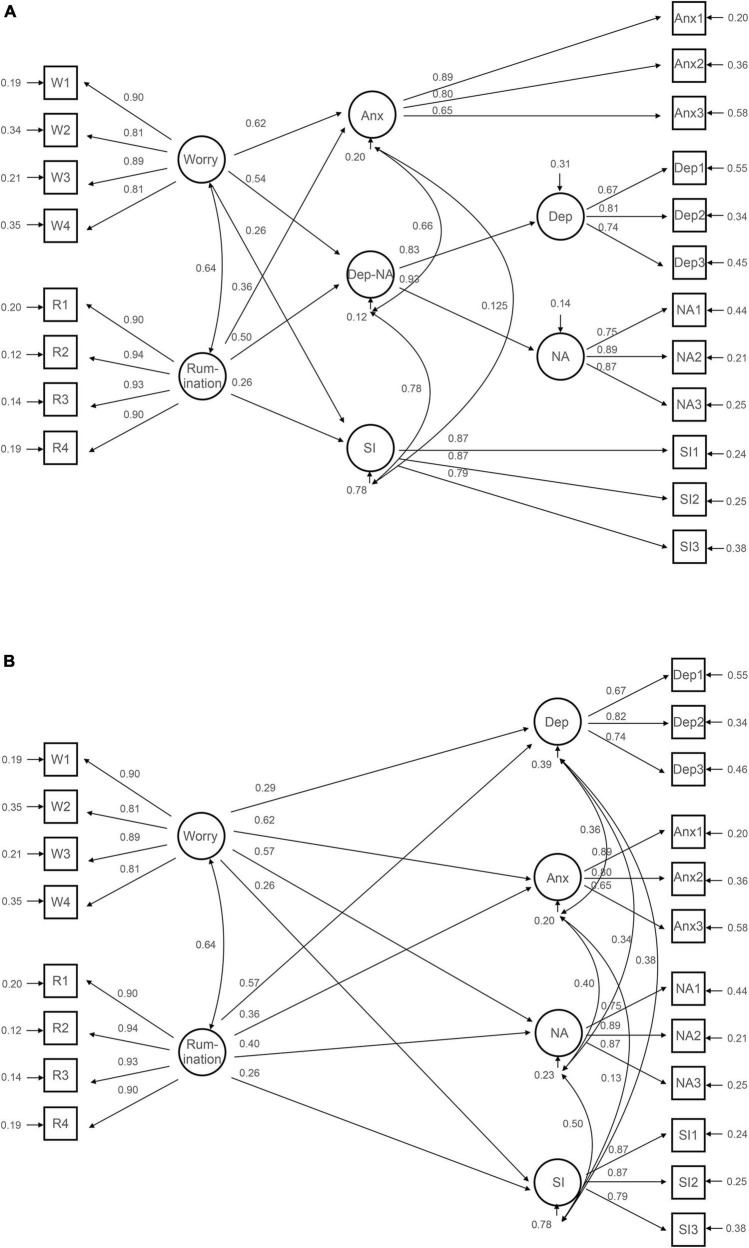
Structural equation modeling of the relationship between depression, anxiety, type D personality, worry and rumination. The figures shows standardised coefficients. The circles represent latent variables and the squares represent observed variables. **(A)** Shows the relationship between Worry and Rumination and the factors Anx, Dep-NA and SI. **(B)** Shows the relationsship between Worry and Rumination and the factors Dep, Anx, NA and SI. Anx, Anxiety; Dep, Depression; NA, Negative Affectivity; SI, Social Inhibition.

Results from these analyses (Figure 3A) showed worry to be significantly associated with anxiety (β = 0.62, *p* < 0.001), the Dep-NA factor (β = 0.54, *p* < 0.001), and the social inhibition factor (β = 0.26, *p* < 0,001). Rumination was significantly associated with the Dep-NA factor (β = 0.50, *p* < 0.001), anxiety (β = 0.36, *p* < 0.001) and the social inhibition factor (β = 0.26, *p* < 0.001) (Figure 3A). Similar results were found in SEM Model 2, as illustrated in Figure 3B.

Both models showed strong associations between worry and anxiety, and moderate associations either between rumination and depression and negative affectivity, or moderate associations between rumination and the latent factor underlying depression and negative affectivity (Dep-NA). Both structural models yielded acceptable goodness of fit indices (for the model with Dep-NA as outcomes: χ^2^ = 597.4, *df* = 158, SRMR = 0.033, RMSEA = 0.055, CFI = 0.964, TLI = 0.957; for the model with all HADS and DS14 factors as outcomes; χ^2^ = 569.2, *df* = 155, SRMR = 0.031, RMSEA = 0.054, CFI = 0.966, TLI = 0.959).

## Discussion

The existing literature suggests that there might be conceptual similarities between the constructs of depression, anxiety and negative affectivity that could be accounted for by a common denominator. One goal of this study was to shed light on this discrepancy. To the best of our knowledge, this is the first study to explore the potential overlap between the constructs of depression and anxiety as measured by the HADS, and the negative affectivity and social inhibition constructs as measured by the DS14 and how these constructs are associated with worry and rumination in outpatients with chronic CHD. First, the results suggested an overlap between the negative affectivity factor of DS14 and the depression factor of HADS. This finding indicates that these two constructs share some conceptual and measurement properties. Second, our results showed that worry and rumination were differentially associated with depression, anxiety, and negative affectivity. Consistent with the metacognitive model (Wells, 2009), the data suggest that intervention efforts could benefit from targeting worry and/or rumination in the treatment of CHD outpatients with symptoms of psychological distress.

### Is there an overlap between the constructs of psychological distress?

We found a good fit for several models describing the degree of overlap between the scales of the HADS and the DS14 instruments in patients with chronic CHD. Based on previous findings (Ossola et al., 2015), we hypothesized and tested the possibility of an overlap between the depression factor of HADS and the negative affectivity factor of DS14 (Dep-NA). Results based on the overall fit indices largely indicated good fit for this model, suggesting that there could be an underlying phenomenon or dimension that reflects common variance (i.e., overlap) between these two factors. The baseline model, where the four latent factors depression, anxiety, negative affectivity, and social inhibition were estimated as distinct, independent factors, also showed good fit to the data. However, based on the fit indices, it appears that the Dep-NA model reflecting overlap between the depression factor of HADS and negative affectivity factor of DS14 is a more parsimonious model than the baseline model. Yet, these results should be interpreted with caution, as our models were non-nested and thus not eligible for tests of model fit.

Notwithstanding, these results are in line with findings from previous studies suggesting that negative affectivity is strongly correlated with depressive symptoms (Starrenburg et al., 2013; Conden et al., 2014), and with the results reported in Ossola et al. (2015) study. However, our results add to these previous findings by indicating that there is substantial construct and measurement overlap between the negative affectivity factor of DS14 and the depression factor of HADS at the factor level, using a higher-order CFA approach which is superior at ruling out measurement error. In this sense, this study adds important knowledge that compliments and further elaborates the findings of the Ossola study, which analyzed potential overlap at item-level. Moreover, our findings are in contrast with those of Kudielka et al. (2004) and Pelle et al. (2009) studies, which found little or no overlap between DS14-negative affectivity and the items of the scales of HADS.

### Is there overlap between the social inhibition and negative affectivity scales of the DS14?

Our findings did not support the hypothesis of overlap between the social inhibition and the negative affectivity scales of the TDP instrument. This is in line with what has been previously found regarding the relationship between negative affectivity and social inhibition—that little variance is shared between these two factors which, in turn, indicates that they represent relatively distinct and independent constructs (Ossola et al., 2015). The weaker correlations found between the social inhibition factor and the anxiety and depression factors are also in line with previous factor analytic studies reporting little or no correlation between social inhibition and other forms of psychological distress (Denollet and Brutsaert, 1998; Kudielka et al., 2004; Pelle et al., 2009; Svansdottir et al., 2012). In this sense, our findings are consistent with those reported in Denollet and Brutsaert (1998) study where principal component analysis was used to explore potential overlap between several indicators of psychological distress (negative affectivity, social inhibition, anxiety, pessimism, despair, and anger) and found support for two higher order factors that differentiated negative affectivity and the other indicators of psychological distress from social inhibition. Thus, in general, little support has been found for an overlap between the negative affectivity and social inhibition factors of the DS14 instrument in the limited number of studies assessing the nature of relations between these factors (Kudielka et al., 2004; Pelle et al., 2009; Svansdottir et al., 2012; Ossola et al., 2015). More specifically, three of these studies concluded that negative affectivity and social inhibition appear to be distinct entities (Kudielka et al., 2004; Pelle et al., 2009; Svansdottir et al., 2012). In total, our results together with the results of these previous studies strongly support that negative affectivity and social inhibition may represent different constructs rather than one overarching TDP factor.

### Discrepancies between the current study and previous studies

A possible explanation of the discrepancies between our findings of an overlap between negative affectivity and depression and the results of previous studies contradicting this finding could be the differences in population characteristics. We do not know if the relationship between the investigated factors differs across characteristics such as age, gender, and comorbid conditions including CHD. Our results are in line with those of Ossola et al. (2015) study conducted in CHD patients. As another study using a relatively young sample of healthy men (Kudielka et al., 2004) yielded different results, it may suggest that sample characteristics is of importance. Another explanation of the discrepancies in results could be that there is a key dimension that has not been taken into consideration in previously tested models. For instance, Segerstrom et al. (2000) suggested that repetitive negative thinking (i.e., worry and rumination) could be a concomitant of anxiety and depression. Nordahl et al. (2018) speculated about the existence of a general vulnerability factor that might foster anxiety and/or depression, and they found that metacognitive beliefs (i.e., beliefs about thoughts and cognitive processes), may represent such a vulnerability factor.

### How are the constructs of depression, anxiety, negative affectivity, social inhibition associated with worry and rumination?

Results from our regression analyses showed that the latent factors of depression, anxiety, negative affectivity, and social inhibition were moderately to strongly correlated with worry and rumination. Both SEM models showed a significant and moderate to strong association between worry, rumination and the depression and anxiety factors as well as the negative affectivity factor. Consistent with previous studies on the relationship between worry and rumination with depression and anxiety (Wahl et al., 2019), we found that worry had a stronger association with anxiety whilst rumination had a stronger association with depression. Despite their conceptual similarities and some previous arguments in support of common processes (Ehring and Watkins, 2008), previous studies have emphasized the differences between worry and rumination (Papageorgiou and Wells, 1999) as well as their important relationships to depression and anxiety (Goring and Papageorgiou, 2007). We also found a moderate association between worry and negative affectivity and the Dep-NA-factor, despite a weak association between worry and depression. Noteworthy, only small associations were found between social inhibition and worry and rumination. Therefore, a change in the level of worry or rumination may potentially be expected to yield significant changes in levels of depression, anxiety and negative affectivity, but not in the level of social inhibition.

### Future directions and clinical implications

The results that higher levels of anxiety were predicted by higher levels of worry and that higher levels of rumination predicted higher levels of depression and negative affectivity are in line with the metacognitive model of anxiety and depression (Wells, 2009). The psychological treatment derived from this model, namely MCT, has recently been found to be effective for the treatment of symptoms of depression and anxiety among CHD patients in a cardiac rehabilitation setting after an acute event (Wells et al., 2021). However, the results for specific changes in worry and rumination were not reported. Nonetheless, studies have been conducted in other patients that have reported worry and rumination to change in parallel with a decrease in symptoms of depression and anxiety (Solem et al., 2019). We are also aware of other treatment efforts have been made at modifying worrisome and ruminative thinking (Querstret and Cropley, 2013; Watkins, 2015; Monteregge et al., 2020). Our current results suggest that efforts aimed at reducing worry and rumination may reduce psychological distress such as anxiety, depression, and negative affectivity in patients with CHD. Therefore, our results encourage further testing of the metacognitive model and therapy in CHD patients. It would be particularly important to test the effect on negative affectivity and its consequences for type D status because yet there is no effective therapy for the type D personality and its factors (Raykh et al., 2022) even though the most recent study reports an association between TDP and negative cardiovascular prognosis in CHD patients.

### Strengths and limitations

Limitations include the exclusion of some participants due to missing responses in their questionnaires, but we did not find any significant differences between the total sample and the 1,042 responders of HADS and DS14 or between the HADS/TDP sample and the 904 responders of HADS, DS14, PSWQ, and RRS. We did not assess other potentially key underlying vulnerability factors such as metacognitions. Other potential important factors such as anger and insomnia were not included. Our sample did not allow for gender-specific analyses that may be of importance, since there is some evidence that emotional regulation of female CHD patients may differ from those of male patients (Kubzansky and Thurston, 2007). Our results are based on assessment by the HADS for anxiety and depression. We do not know how measurements with other instruments for anxiety and depression could impact on the results, e.g., a measure of social anxiety might be more strongly correlated with social inhibition (Kupper and Denollet, 2014). The strengths of this study include the catchment area being representative for the Norwegian population, when considering the sociodemographic and clinically relevant factors (Munkhaugen et al., 2016), a high participation rate (83%) and consecutive recruitment of patients from routine practice from two general hospitals. These strengths ensured a clinically representative CHD outpatient study group albeit a survival bias may be present in the study population. One hundred and sixty patients died between the cardiac event and inclusion to the study, and these patients may have been in a poorer clinical and psychosocial condition compared to those patients already included. In this respect, inclusion of these patients could perhaps have led to unknown differences in the results.

## Conclusion

Our findings indicate that there may be an underlying dimension between some of the factors of the HADS and the DS14 instruments, particularly the depression factor of HADS and the NA factor of DS14. Worry and rumination were significantly associated with depression, anxiety, and negative affectivity. Therefore, our results suggest that therapeutic methods such as MCT, which target worry and rumination, could be effective for CHD patients with significant symptoms of depression, anxiety and/or negative affectivity, and should be further investigated.

## Data availability statement

According to Norwegian legislation, the Norwegian Data Protection Authority, and the Committee of Ethics, we are not allowed to share original study data publicly. However, the essential generated data are available from the corresponding author on reasonable request.

## Ethics statement

The studies involving human participants were reviewed and approved by the Regional Committees for Medical Research Ethics South East Norway. The patients/participants provided their written informed consent to participate in this study.

## Author contributions

TD, CP, KT, and SB contributed to the idea and design of the study. JM contributed to the data collection and scoring. KT and SB contributed to the data analysis and interpretation. KT was responsible for the first draft of the manuscript. All authors contributed significantly to the final version of the manuscript.
