# Supplementary material for: Relationships between depression, anxiety, type D personality, and worry and rumination in patients with coronary heart disease
Source: Front Psychol. 2022 Sep 14;13:929410. doi: 10.3389/fpsyg.2022.929410 (PMC9517376; doi:10.3389/fpsyg.2022.929410)
Supplement: Supplementary file 1 [file Data_Sheet_1.docx]

**Supplementary tables**

**Supplementary table 1 − Comparison of clinical characteristics in the total sample (n=1127), responders of HADS and DS14 (n=1042) and responders of HADS, DS14, PSWQ and RRS (n=904):**

|  | **Total sample** | **Full responders of HADS and DS14** | **Full responders of HADS, DS14, PSWQ and RRS** | **P-value. Total sample versus responders of HADS and DS14** | **P-value. Total sample versus responders of HADS, DS14, PSWQ and RRS** |
| --- | --- | --- | --- | --- | --- |
| **Age at event, mean (SD)** | 61.6 (9.6) | 61.5 (9.6) | 61.1 (9.6) | 0.83 | 0.23 |
| **Gender (by female gender), % (n)** | 21.0 (237) | 20.8 (217) | 18.9 (171) | 0.91 | 0.24 |
| **Education (Less than university/college), % (n)** | 70.3 (780) | 70.2 (725) | 68.6 (617) | 0.99 | 0.43 |
| **Time since event (months), mean (SD)** | 17.1 (10.5) | 17.2 (10.5) | 17.2 (10.6) | 0.70 | 0.75 |
| **Acute myocardial infarction, % (n)** | 79.5 (896) | 79.1 (824) | 78.0 (705) | 0.81 | 0.41 |
| **Stable or unstable angina, % (n)** | 20.5 (231) | 20.9 (218) | 22.0 (199) | 0.81 | 0.41 |
| **Previously one or more coronary event prior to the index event, % (n)** | 23.7 (267) | 24.1 (251) | 23.6 (213) | 0.83 | 0.95 |
| **Participation in cardiac rehabilitation, % (n)** | 50.3 (567) | 51.1 (532) | 52.0 (470) | 0.73 | 0.45 |
| **CRP more or equal to 2 mg/L, % (n)** | 40.6 (430) | 39.9 (392) | 38.5 (338) | 0.73 | 0.59 |
| **Charlston co-morbidity score, mean (SD)** | 4.1 (1.4) | 4.1 (1.4) | 4.0 (1.4) | 0.64 | 0.10 |
| **Type D personality, % (n)** | 18.0 (197) | 18.1 (189) | 18.4 (166) | 0.93 | 0.90 |
| **Low density lipoprotein cholesterol >1.8 mmol/L, % (n)** | 58.6 (660) | 58.3 (607) | 57.9 (523) | 0.88 | 0.75 |
| **Smoking by event, % (n)** | 34.6 (390) | 34.1 (355) | 34.1 (308) | 0.79 | 0.80 |
| **Daily smoker by inclusion, % (n)** | 21.2 (230) | 2.3 (204) | 20.1 (176) | 0.61 | 0.54 |
| **Diabetes, % (n)** | 23.4 (257) | 23.3 (237) | 23.6 (209) | 0.95 | 0.93 |
| **Physical activity < 1 times per week, % (n)** | 18.0 (197) | 17.2 (176) | 15.6 (138) | 0.65 | 0.16 |
| **Blood pressure > 140/90 (140/80 diabetes) mmHg, % (n)** | 45.2 (457) | 45.1 (415) | 44.0 (366) | 0.99 | 0.63 |
| **Body Mass Index >30 kg/m2, % (n)** | 33.6 (340) | 33.4 (315) | 33.7 (280) | 0.94 | 0.97 |
| **Antithrombotic, % (n)** | 97.3 (1096) | 97.6 (1017) | 97.9 (885) | 0.61 | 0.35 |
| **Statins, % (n)** | 92.6 (1043) | 92.9 (968) | 93.7 (847) | 0.75 | 0.31 |

*HADS, Hospital Anxiety and Depression Scale; DS14, Type D Scale; PSWQ, Penn State Worry Questionnaire; RRS, Ruminative Responsive Scale.*

**Supplementary table 2 − Fit Indices for the Measurement Models:**

|  | **Degrees of freedom** | **χ2** | **SRMR** | **RMSEA** | **CFI** | **TLI** |
| --- | --- | --- | --- | --- | --- | --- |
| **HADS-A** | 0 | 0 | 0 | 0 | 1 | 1 |
| **HADS-D** | 0 | 0 | 0 | 0 | 1 | 1 |
| **DS14-NA** | 0 | 0 | 0 | 0 | 1 | 1 |
| **DS14-SI** | 0 | 0 | 0 | 0 | 1 | 1 |
| **PSWQ** | 2 | 70.7 | 0.026 | 0.195 [95% CI: 0.158 – 0.235] | 0.971 | 0.914 |
| **RRS** | 2 | 34.4 | 0.011 | 0,134 [95% CI: 0.097 – 0.175] | 0.986 | 0.957 |

*HADS-A, Hospital Anxiety and Depression Scale anxiety subscale; HADS-D, Hospital Anxiety and Depression Scale depression subscale; DS14 NA, Type D Scale negative affectivity subscale; DS14 SI, Type D Scale social inhibition subscale; PSWQ, Penn State Worry Questionnaire; RRS, Ruminative Responsive Scale,* χ^2^*: Chi-square test, SRMR: Standardised Root Mean Squared Residual, RMSEA: Root Mean Square Error of Approximation, CFI: Comparative Fit Index, TLI: Tucker-Lewis Index.*
